# Sphingosine 1-Phosphate Mediates Hyperalgesia via a Neutrophil-Dependent Mechanism

**DOI:** 10.1371/journal.pone.0055255

**Published:** 2013-01-25

**Authors:** Amanda Finley, Zhoumou Chen, Emanuela Esposito, Salvatore Cuzzocrea, Roger Sabbadini, Daniela Salvemini

**Affiliations:** 1 Department of Pharmacological and Physiological Science, Saint Louis University School of Medicine, St. Louis, Missouri, United States of America; 2 Department of Clinical and Experimental Medicine and Pharmacology, University of Messina, Messina, Italy; 3 Lpath, Inc., and Department of Biology, San Diego State University, San Diego, California, United States of America; University of Cincinnati, United States of America

## Abstract

Novel classes of pain-relieving molecules are needed to fill the void between non-steroidal anti-inflammatory agents and narcotics. We have recently shown that intraplantar administration of sphingosine 1-phosphate (S1P) in rats causes peripheral sensitization and hyperalgesia through the S1P_1_ receptor subtype (S1PR_1_): the mechanism(s) involved are largely unknown and were thus explored in the present study. Intraplantar injection of carrageenan in rats led to a time-dependent development of thermal hyperalgesia that was associated with pronounced edema and infiltration of neutrophils in paw tissues. Inhibition of 1) S1P formation with SK-I, a sphingosine kinase inhibitor, 2) S1P bioavailability with the S1P blocking antibody Sphingomab, LT1002 (but not its negative control, LT1017) or 3) S1P actions through S1PR_1_ with the selective S1PR_1_ antagonist, W146 (but not its inactive enantiomer, W140) blocked thermal hyperalgesia and infiltration of neutrophils. Taken together, these findings identify S1P as an important contributor to inflammatory pain acting through S1PR_1_ to elicit hyperalgesia in a neutrophil-dependant manner. In addition and in further support, we demonstrate that the development of thermal hyperalgesia following intraplantar injection of S1P or SEW2871 (an S1PR_1_ agonist) was also associated with neutrophilic infiltration in paw tissues as these events were attenuated by fucoidan, an inhibitor of neutrophilic infiltration. Importantly, FTY720, an FDA-approved S1P receptor modulator known to block S1P-S1PR_1_ signaling, attenuated carrageenan-induced thermal hyperalgesia and associated neutrophil infiltration. Targeting the S1P/S1PR_1_ axis opens a therapeutic strategy for the development of novel non-narcotic anti-hyperalgesic agents.

## Introduction

One-quarter of Americans over the age of 20 suffer from some sort of persistent pain [1]. Current treatment options, such as non-steroidal anti-inflammatory agents and narcotics, result in deleterious side-effects making them unattractive options for persistent use [2]. Therefore, novel classes of pain-relievers are severely needed. In addition to their pro-inflammatory roles [3], sphingolipids including ceramide [4]–[10] and sphingosine 1-phosphate (S1P) [6], [7], [10]–[15] are emerging as important modulators of pain.

S1P derived from the conversion of ceramide to sphingosine by ceramidase, and is a product of the phosphorylation of sphingosine by sphingosine kinase isoenzymes, plays an important role in peripheral and central sensitization. S1P resulting from ceramide bioconversion has been shown to contribute to NGF-induced excitation of rat sensory neurons [11] and is required for the development of ceramide-induced peripheral sensitization following intraplantar injection of ceramide in rats [7]. Furthermore, S1P has the ability to directly increase the excitability of rat sensory neurons in vitro [14] and cause thermal hyperalgesia following intraplantar injection in rats [12]. However, apart from S1P's ability to directly increase nociceptor sensitivity *in vitro* and *in vivo* [13] and our previous reports that S1P exerts its actions at least in part via the upregulation of peroxynitrite [12], S1P's mechanism of action remains largely uninvestigated.

To date, five subtypes of G-protein coupled S1P receptors (S1PR_s_) have been identified: S1PR_1–5_ [16]. These receptors are differentially expressed on all cell types and can bind to multiple different heterotrimeric G-proteins [16], [17], thereby having varying effects, depending on the signaling cascade they activate. In order to examine the signaling pathways and mechanisms involved in S1P-mediated hyperalgesia it is important to identify the receptor subtype(s) involved. We have focused our studies on S1PR_1_ as we have shown this receptor subtype to be of particular importance in S1P-mediated peripheral hyperalgesia [12]. In addition, enhanced excitability in peripheral sensory neurons in response to S1P been shown to occur, at least in part, through the activation of S1PR_1_ [18] and S1P hypersensitivity is significantly reduced in mice with a conditional nociceptor-specific deletion of S1PR_1_ [13] or those with local knockdown of S1PR_1_ in the DRG [19].

Another important action of S1P is its ability to enhance immune cell migration [20]. Specifically, S1P via activation of S1PR_1_ upregulates the expression of the adhesion molecules ICAM-1 and E-selectin on the surface of endothelial cells [21]–[24] thereby initiating neutrophilic infiltration [25], [26]. Many models of inflammation-induced hyperalgesia rely on neutrophilic recruitment [27]–[30] and this neutrophil-dependent hyperalgesia underlies pain of several etiologies.

Taken together, we hypothesize and demonstrate herein that neutrophils contribute to the development of S1P-induced hyperalgesia acting through the S1PR_1_ subtype. Targeting the S1P-to-S1PR_1_ pathway may offer a novel approach in the management of pain.

## Materials and Methods

### Materials

S1P (sphingosine 1-phospate), W146 (3- amino- 4- (3- hexylphenylamino)- 4- oxobutyl phosphonic acid), W140 (3- amino- 4- (3- hexylphenylamino)- 4- oxobutyl phosphonic acid), JTE-013 (N- (2, 6- dichloro- 4- pyridinyl)- 2- [1, 3- dimethyl- 4- (1- methylethyl)- 1H- pyrazolo[3, 4- b]pyridin- 6- yl]- hydrazinecarboxamide), CAY10444 (2- undecyl- thiazolidine- 4- carboxylic acid) and FTY720 (2- amino- 2- [2- (4- octylphenyl)ethyl]- 1, 3- propanediol, hydrochloride) were all purchased from Cayman Chemical (Ann Arbor, MI). Carrageenan, fucoidan and o-dianisidine (3,3′-Dimethoxybenzidine dihydrochloride) were purchased from Sigma-Aldrich (St. Louis, MO). The SK inhibitor, SK-I [2-(*p*-hydroxyanilino)-4-(*p*-chlorophenyl) thiazole] was purchased from Calbiochem (La Jolla, CA) and the bicinchoninic acid (BCA) assay was from Thermo-Fisher (Rockford IL). The murine monoclonal anti-S1P antibody, Sphingomab/LT1002 and its isotype mAb control, LT1017, was generated as described previously [31].

### Animals

Male Sprague Dawley rats (200–220 g) were purchased from Harlan (USA) and housed 3–4 per cage and maintained in a controlled environment (12 h light/dark cycle) with food and water available *ad libitum*. All experiments were performed in accordance with the International Association for the Study of Pain and the National Institutes of Health guidelines on laboratory animal welfare and the recommendations by Saint Louis University Institutional Animal Care and Use Committee.

### Drug Administration

Male Sprague Dawley rats were lightly anesthetized [CO_2_ (80%)/O_2_ (20%)] and given a subplantar injection of S1P (0.3 µg; using a Hamilton gauge needle 3 ½”; 5 µL) or of 1% carrageenan (100 µL) into the left hindpaw. All drugs or their vehicle (6% EtOH in saline for S1P; saline for carrageenan) were given by intraplantar injection 30 minutes prior to intraplantar S1P or carrageenan injection unless otherwise stated. LT1002 and LT1017 were given in a volume of 40 µL while SK-I, W146, W140, JTE-013 and CAY10444 were given in a volume of 5 µL. Fucoidan was given i.p. in 200 µL saline, 30 minutes prior to S1P injection. FTY720 was given p.o. in 10% DMSO in saline, 30 min prior to carrageenan injection.

### Behavioral Analysis

Behavioral testing was done with experimenter blinded to treatment conditions. Hyperalgesic responses to heat were determined by the Hargreaves' Method using a Basile Plantar Test [32] with a cut-off latency of 20 s employed to prevent tissue damage. Rats were individually confined to plexiglass chambers and allowed to habituate. A mobile unit consisting of a high intensity projector bulb was positioned to deliver a thermal stimulus directly to an individual hindpaw from beneath the chamber. The withdrawal latency period of injected paws was determined with an electronic clock circuit and thermocouple. Results are expressed as paw-withdrawal latency(s).

### Carrageenan-Induced Paw Edema

Changes in paw volume were measured as previously described [33]. Briefly, paw volume was measured with a plethysmometer (Ugo Basile, Comerio, Varese, Italy) immediately prior to the injection of carrageenan and thereafter at hourly intervals for 6 h. Edema was expressed as the increase in paw volume (mL) after carrageenan injection relative to the pre-injection value for each animal. Results are expressed as paw volume change (mL).

### Histological Examination

For histopathological examination, biopsies of paws were taken 2 hours following the intraplantar injection of carrageenan, tissue from the pads of the rats hindpaw was removed with a scalpel. The tissue slices were fixed in Dietric solution (14.25% ethanol, 1.85% formaldehyde, 1% acetic acid) for 1 week at room-temperature, dehydrated by graded ethanol and embedded in Paraplast (Sherwood Medical). Section (thickness 7 µm) were deparaffinized with xylene, stained with hematoxylin and eosin and observed in Dialux 22 Leitz microscope.

### Myeloperoxidase Assay

Myeloperoxidase (MPO; a peroxidase enzyme released by neutrophils and a marker of neutrophilic infiltration [34], [35]) activity was assessed by taking tissue at 2 h (time of peak inhibition). Flash-frozen plantar soft tissue was pulverized in liquid nitrogen-chilled mortar and pestle, and then homogenized in 1 mL 0.05% HTAB in 50 mM potassium phosphate buffer and kept on ice. Homogenates were sonicated with an ultrasonicator for 5×10 s, centrifuged 40,000 g @ 4°C for 15 min, then supernatants were pulled off and stored at 4°C. For the assay, 7 µL of sample was added to 193 uL of 0.167 mg/mL o-dianisidine in 50 mM potassium phosphate buffer with or without 0.0005% H_2_O_2_. Absorbance of each sample was read immediately and at 1 min intervals for 3 min at 460 nm. To calculate MPO activity, we plotted absorbance over time to obtain slope and used slope to calculate units of activity per mg (U/mg) using the equation U/mg  =  (ΔA_460_/min)/(11.3× mg enzyme/ml reaction mixture).

### Statistical Analysis

Differences in thermal hyperalgesia were assessed using two-way analysis of variance (ANOVA) with Bonferroni post hoc comparisons to S1P or carrageenan-treated animals. Differences in MPO activity levels were assessed by one-way ANOVA followed by Dunnett's post hoc comparisons to S1P or carrageenan-treated animals. Differences in paw volume were analyzed by using student's unpaired *t* test. Significant statistical difference was defined when P-value <0.05.

## Results

### Carrageenan-induced thermal hyperalgesia is associated with an increase in neutrophilic recruitment which is blocked by fucoidan

**Figure 1:**
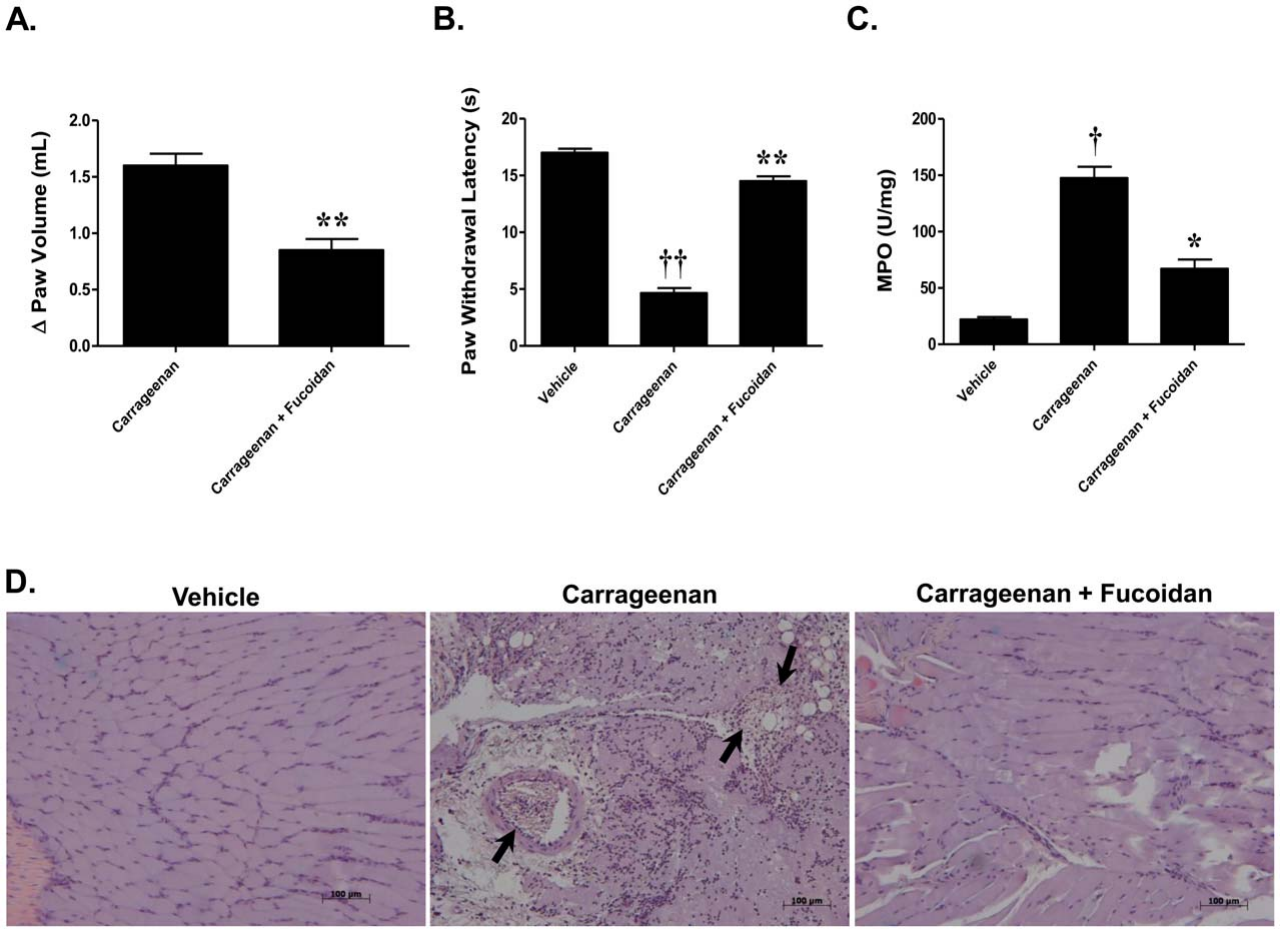
Carrageenan injection leads to an increase in neutrophil infiltration that is attenuated by fucoidan. A–C) Intraplantar injection of carrageenan (1%) led to a time-dependent development of thermal hyperalgesia that was accompanied by an increase in paw volume and an increase in myeloperoxidase activity. All were blocked by fucoidan (40 mg/kg). D) The increased myeloperoxidase activity in response to carrageenan injection correlated with pathological changes as well as a marked increase in neutrophilic infiltration as indicated by H&E staining. Fucoidan (40 mg/kg) attenuated this response. Results are expressed as mean ± SEM for 6 rats and analyzed by student's unpaired *t* test for paw volume, two-way repeated measures ANOVA with Bonferroni *post hoc* test for behavior and one-way ANOVA with Dunnett's *post hoc* test for MPO, where *P<0.01, **P<0.001 *vs*. carrageenan; † *P*<0.01, †† *P*<0.001 *vs*. vehicle.

The carrageenan model is a well-characterized model of inflammation-induced thermal hyperalgesia which has been suggested to rely on neutrophilic infiltration [28]. The development of edema and thermal hyperalgesia in response to intraplantar injection of carrageenan (1%, n = 6) seen at peak (6 h) was associated with increased infiltration of neutrophils as shown by an increase in myeloperoxidase activity (MPO; a peroxidase enzyme released by neutrophils and a marker of neutrophilic infiltration [34], [35]) and by histological examination of paw tissues (Figure 1). Administration of fucoidan (40 mg/kg, n = 6), a well- characterized P- and L-selectin blocker, that is well established in the literature as a potent inhibitor of neutrophil adhesion, rolling and infiltration at inflammatory sites [28], [36], [37], prevented the edema associated with carrageenan injection (Figure 1A), blocked the thermal hyperalgesia (Figure 1B) and significantly reduced myeloperoxidase activity (Figure 1C). Upon histological examination, the paws revealed pathologic changes that correlated closely with the increases in MPO activity. Paw biopsies showed that after carrageenan administration, marked inflammatory changes were observed including pronounced neutrophil infiltration (Figure 1D, see arrows). Treatment with fucoidan significantly reduced overall pathological changes and neutrophil infiltration in the paw tissues (Figure 1D).

### Blocking S1P inhibits carrageenan-induced thermal hyperalgesia

**Figure 2:**
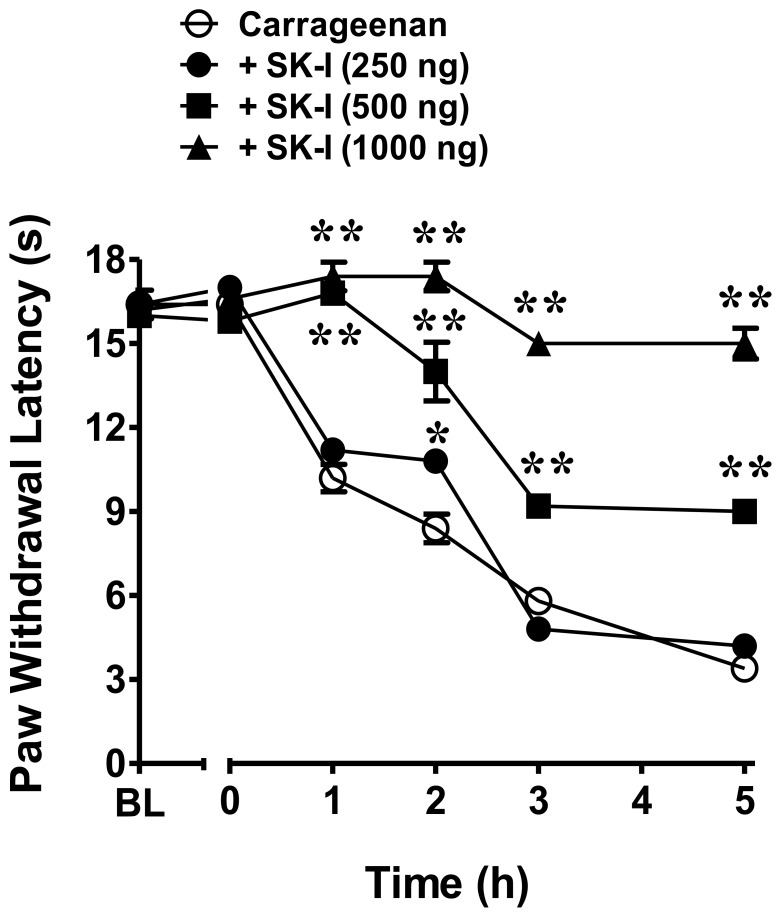
Carrageenan-induced thermal hyperalgesia is blocked by SK-I. Intraplantar injection of carrageenan (1%) led to a time-dependent development of thermal hyperalgesia that was attenuated in a dose-dependent manner by intraplantar injection of SK-I (250 ng, 500 ng, or 1000 ng; n = 6). Results are expressed as mean ± SEM and analyzed by two-way repeated measures ANOVA with Bonferroni *post hoc* test where *P<0.01, **P<0.001 *vs.* carrageenan.

**Figure 3:**
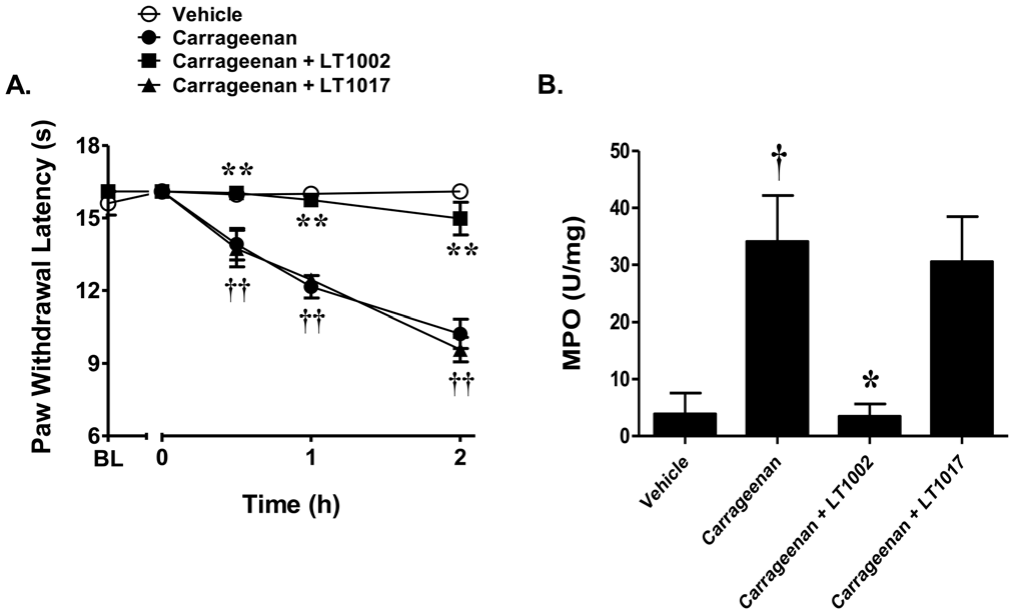
Inhibition of S1P attenuates carrageenan-induced thermal hyperalgesia and the recruitment of neutrophils. A) Intraplantar injection of LT1002 (484 μg, n = 6) but not of LT1017 (572 μg; isotype control, n = 6) attenuated carrageenan-induced thermal hyperalgesia. B) Intraplantar injection of carrageenan led to an increase in neutrophilic recruitment as evidenced by increased levels of MPO activity in paw tissues and this was blocked by LT1002 but not LT1017. Results are expressed as mean ± SEM and analyzed by two-way repeated measures ANOVA with Bonferroni *post hoc* test for behavior and one-way ANOVA with Dunnett's *post hoc* test for MPO, where *P<0.01, **P<0.001 *vs.* carrageenan; † *P*<0.05, †† *P*<0.001 *vs.* vehicle.

Intraplantar injection of carrageenan led to a time-dependent development of thermal hyperalgesia that peaked at 3 h and was sustained through 5 h (Figure 2). Intraplantar injection of SK-I, a well-characterized, competitive and reversible inhibitor of sphingosine kinase, and thus S1P [38], given 30 minutes before carrageenan, inhibited the development of carrageenan-induced thermal hyperalgesia in a dose-dependent manner (250–1000 ng, n = 6, Figure 2). Doses were chosen from previous studies [38]–[41]. Similarly, treatment with LT1002 (484 μg, n = 6), a monoclonal antibody directed against S1P [31], was able to significantly attenuate the carrageenan-induced hyperalgesic response, while its IgG1κ isotype control, LT1017 (572 μg, n = 6), had no effect (Figure 3A). These results suggest that S1P contributes to the development of carrageenan-induced thermal hyperalgesia.

### Inhibition of S1P blocks the increased neutrophilic recruitment associated with carrageenan-induced thermal hyperalgesia

To determine whether S1P mediates the recruitment of neutrophils in carrageenan-induced thermal hyperalgesia, plantar tissues were taken from animals at 2 h (time of peak inhibition, data not shown) and assayed for MPO activity. As can be seen in Figure 3B, carrageenan injection led to a significant increase in MPO activity that was completely abrogated by pretreatment with LT1002 (484 μg, n = 6), but not by its negative control, LT1017 (572 μg, n = 6).

### Blocking the S1P-S1PR_1_ axis attenuates carrageenan-induced thermal hyperalgesia and neutrophilic recruitment

**Figure 4:**
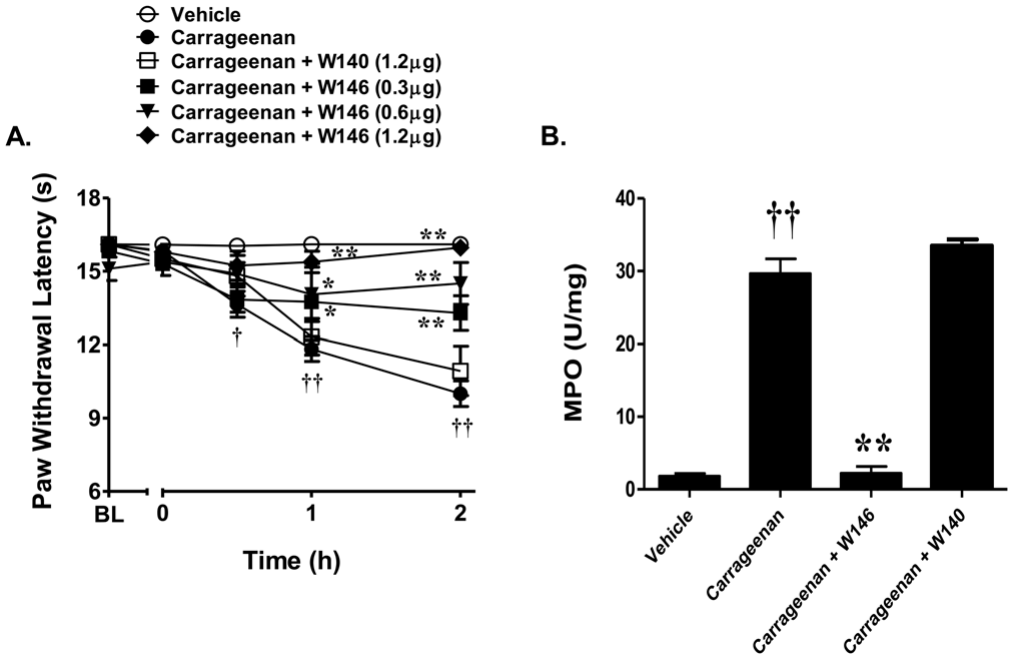
Blockade of S1PR_1_ inhibits carrageenan-induced thermal hyperalgesia and the recruitment of neutrophils. A) Intraplantar injection of carrageenan led to a time-dependent development of thermal hyperalgesia that was blocked in a dose-dependent manner by the selective S1PR_1_ antagonist, W146 (0.3–1.2 μg, n = 6) but not by its inactive S-enantiomer, W140 (1.2 µg, n = 6). B) W146 (1.2 µg, n = 6) but not W140 (1.2 µg, n = 6) attenuated neutrophilic recruitment. Results are expressed as mean ± SEM and analyzed by two-way repeated measures ANOVA with Bonferroni *post hoc* test for behavior and one-way ANOVA with Dunnett's *post hoc* test for MPO, where *P<0.05, **P<0.001 *vs*. carrageenan; † *P*<0.01, †† *P*<0.001 *vs*. vehicle.

In order to define whether S1P recruits neutrophils through the S1PR_1_ receptor, we used the well-characterized, selective S1PR_1_ antagonist, W146 [42]. As can be seen in Figure 3, intraplantar injection of W146 (0.3–1.2 μg, n = 6) [43], but not its inactive S-enantiomer, W140 (1.2 μg, n = 6) [43], dose-dependently abrogated the carrageenan-induced hyperalgesic response (Figure 4A). When tested at the highest dose, W146 but not W140 (1.2 μg, n = 6) attenuated neutrophilic recruitment in response to carrageenan (Figure 4B). Doses of W146 and W140 were chosen from previous studies [42].

### S1P and SEW2871-mediated thermal hyperalgesia is attenuated by fucoidan

**Figure 5:**
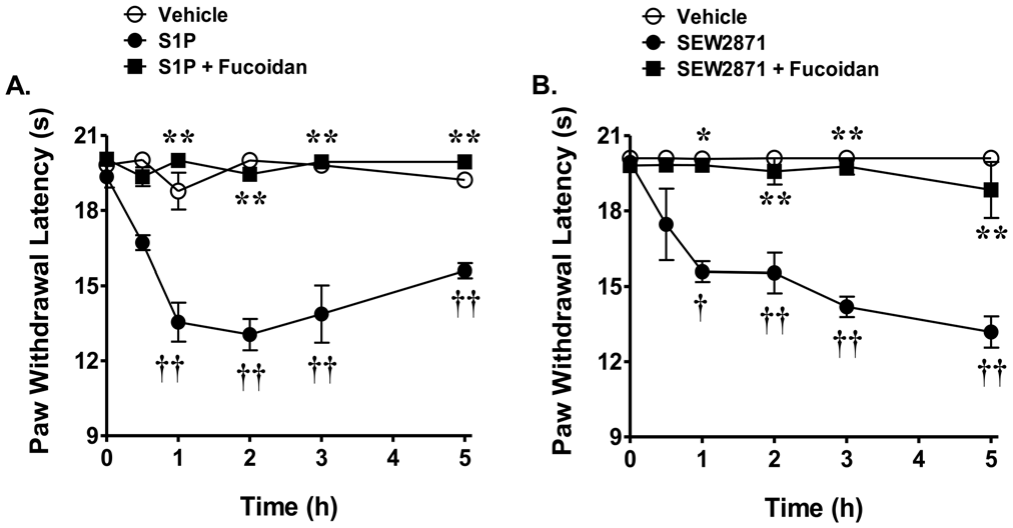
Fucoidan blocks S1P-induced thermal hyperalgesia. Intraplantar injection of A) S1P (0.3 μg) or B) the S1PR_1_ agonist, SEW2871 (0.3 μg), led to a time-dependent development of thermal hyperalgesia that was attenuated by pretreatment with fucoidan (40 mg/kg, i.p.). Results are expressed as mean ± SEM for 6 rats and analyzed by two-way repeated measures ANOVA with Bonferroni *post hoc* test where *P<0.05, **P<0.001 *vs.* carrageenan; † *P*<0.01, †† *P*<0.001 *vs.* vehicle.

To further strengthen the relationship between S1PR_1_ and neutrophil infiltration we investigated whether the development of thermal hyperalgesia in response to exogenous intraplantar injection of S1P or the S1PR_1_ agonist, SEW2871 [43], [44], was driven by neutrophils. As previously reported by our group [7], [12], intraplantar injection of S1P (0.3 μg, n = 6) or SEW2871 (0.3 μg, n = 6) led to a time-dependent development of thermal hyperalgesia (Figure 5) which was blocked by i.p. injection of fucoidan (40 mg/kg, n = 6, Figure 5) given 30 min prior to S1P or SEW2871. S1P and SEW2871 were used at doses previously shown by our group to provide maximal hyperalgesia [7], [12] and were chosen from previous studies [45]. We attempted to measure increased formation of MPO in paw tissues following intraplantar injection of S1P but our results yielded inadequate signal to detect changes in MPO formation between the groups. This may be due to insufficient sensitivity of the assay in these tissues or may have resulted from a highly localized infiltration of neutrophils at sites of damage that is capable of participating in the development of hyperalgesia, but whose signal is undetectable in a total paw preparation. Nevertheless, pharmacological targeting with a well-characterized anti-neutrophil agent [28], [36], [37] clearly supports the contribution of neutrophils in S1P-mediated thermal hyperalgesia.

### FTY720 inhibits carrageenan-induced thermal hyperalgesia and neutrophilic recruitment

**Figure 6:**
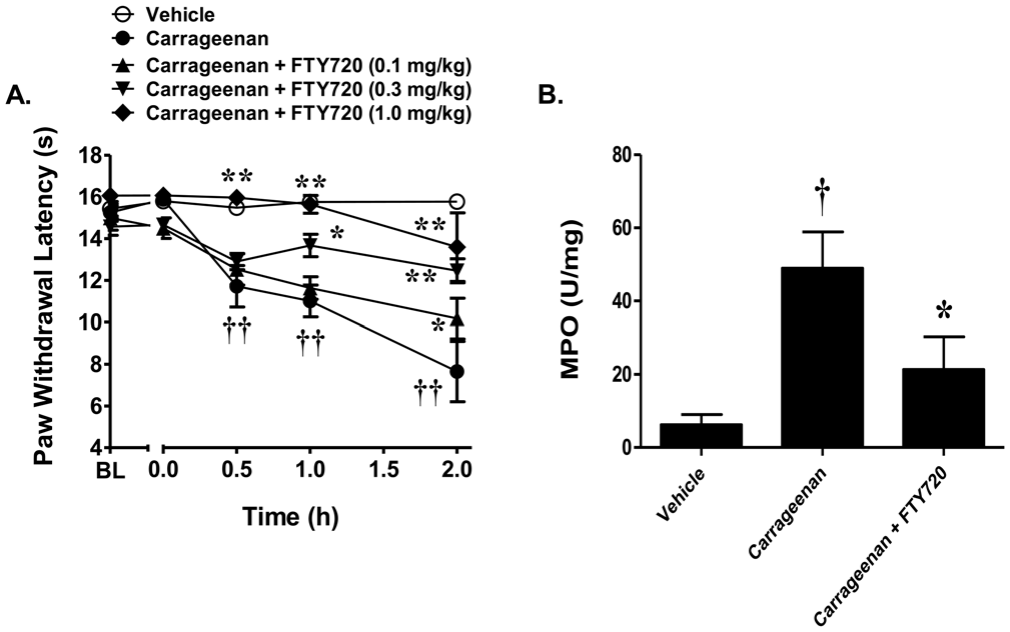
FTY720 attenuates carrageenan-induced thermal hyperalgesia and neutrophilic recruitment. Intraplantar injection of carrageenan (1%) led to a time- dependent development of thermal hyperalgesia. A) Pretreatment with the non-selective functional antagonist, FTY720 (0.1 mg/kg – 1.0 mg/kg, n = 7) attenuated the development of thermal hyperalgesia in response to carrageenan in a dose-dependent manner. B) FTY720 (1 mg/kg, n = 7) attenuated carrageenan-induced increases in neutrophilic recruitment. Results are expressed as mean ± SEM and analyzed by two-way repeated measures ANOVA with Bonferroni *post hoc* test for behavior and one-way ANOVA with Dunnetts *post hoc* test for MPO, where *P<0.05, **P<0.001 *vs*. carrageenan; † *P*<0.01 †† *P*<0.001 *vs*. vehicle.

To assess the therapeutic potential of targeting S1P-S1PR_1_ signaling in the inflammatory pain setting, we examined the ability of the orally active S1PR modulator, FTY720 (fingolimod), to block carrageenan-induced thermal hyperalgesia and neutrophilic recruitment. FTY720 has been recently FDA-approved for the treatment of multiple sclerosis and is postulated to exert its actions, at least in part, through the binding, internalization, and subsequent blockade of S1PR_1_ signaling [46], [47]. Inhibition of S1PR signaling using FTY720 (0.1 mg/kg –1.0 mg/kg, n = 7), with doses chosen from previous studies [46], attenuated the carrageenan-induced hyperalgesia and associated neutrophilic infiltration (Figure 6).

## Discussion

In the present study we demonstrate that S1P acting through the S1P_1_ receptor subtype plays an important role in the development of thermal hyperalgesia associated with inflammation. In addition we present evidence that S1PR_1_-triggered neutrophil infiltration is a central component in this setting. Inhibition of sphingosine kinases 1 and 2 with SK-I, which prevents the phosphorylation of sphingosine to form S1P [38], inhibits the development of thermal hyperalgesia in the carrageenan model, a well-characterized model of inflammation-induced hyperalgesia. Similarly, neutralizing S1P with the anti-S1P blocking antibody, LT1002, prevents the development of the carrageenan hyperalgesic response.

Our present work focuses on the role of S1PR_1_ as it is emerging as an important subtype in the mediation of peripheral sensitization and hyperalgesia. As we have previously reported, blockade of S1PR_1_ with W146 attenuates S1P-induced thermal hyperalgesia [12] and the enhanced excitability in peripheral sensory neurons in response to S1P has been shown to occur at least in part through the activation of S1PR_1_ [18]. It has also been demonstrated that a S1PR_1_ agonist injected intracutaneously induces heat hypersensitivity *in vivo* and that mice lacking S1PR_1_ in Na_v_1.8 expressing nociceptors or in the DRG exhibit reduced S1P-induced hypersensitivity, suggesting that nociceptor sensitization by S1P predominantly occurs through activation of S1PR_1_ [13], [19]. Our results support these previous findings and extend them to also implicate the role for this receptor subtype in inflammatory pain. Indeed, the selective S1PR_1_ antagonist, W146 [43], blocked carrageenan-induced thermal hyperalgesia.

Given that S1P plays a prominent role in the inflammatory process through its ability to recruit neutrophils, which are also implicated in pain [27]–[30], we hypothesized that S1P-induced peripheral sensitization and hyperalgesia may be triggered by neutrophils. In support, we show that carrageenan-induced neutrophil infiltration is dependent upon S1P and subsequent activation of S1PR_1_ as both neutralization of S1P with the anti-S1P mAb, LT1002, and blockade of S1PR_1_ activation with W146 was able to inhibit carrageenan-induced neutrophil infiltration. This evidence, taken with the ability of fucoidan to abrogate the development of thermal hyperalgesia in response to S1P alone, supports our hypothesis that S1P-mediated peripheral sensitization and hyperalgesia occurs via a neutrophil-dependent mechanism.

How neutrophils are recruited at sites of inflammation following activation of the S1P-to-S1PR_1_ pathway remains to be investigated and was not the focus of the present study. However, scientific literature allows us to speculate as to how this might occur. S1PR_1_ activation has been shown to increase the production of the adhesion molecules ICAM-1 and E-selectin in response to inflammatory stimuli, making this a promising candidate for a potential mechanism in our neutrophil-dependent induction of hyperalgesia [22], [48]. Several studies have implicated S1PR_1_ in the activation of the inflammatory transcription factor NFκB and p38 MAP kinase as well [23], [49]. Activation of both NFκB and p38 leads to the increased production of many pro-inflammatory cytokines and chemokines such as TNF-α, IL-1β, IL-6 and CINC-1, the rat homolog of human IL-8 [50]–[52]. These cytokines are known to enhance the migration of neutrophils through their ability to upregulate the expression of adhesion molecules such as ICAM-1 and E-selectin on resident endothelial cells [53] while the chemokine CINC-1 is a potent neutrophil attractant through a mechanism independent of adhesion molecule expression [54]. Interestingly, the potent proinflammatory and pronociceptive nitroxidative species, peroxynitrite [55]–[57], has been shown to play a prominent role in the recruitment of neutrophils in inflammatory conditions, including those induced by carrageenan [58]–[60]. In addition, previous work suggests that peroxynitrite may play a role in the upregulation of the adhesion molecules ICAM-1 and P-selectin as well as the increased production of proinflammatory cytokines such as TNF-α and IL-1β [59], [60]. Taken together with previous work showing that S1P via S1PR_1_ exerts its actions at least in part through the upregulation of peroxynitrite [12], the activation of these signaling pathways elucidates a possible mechanism by which S1PR_1_ may recruit neutrophils to the site of injury.

Whereas this study has clearly demonstrated the role of the S1PR_1_, we are not excluding the potential contribution of other receptor subtypes in S1P's roles; however, this was not the focus of our work. Noteworthy, the tools that are available to examine other receptor subtypes are limited by off-target effects and selectivity issues. For example, the selective S1PR_2_ antagonist, JTE-013, has been shown to actually sensitize sensory neurons independently of S1PR_2_ activation [61]. The S1PR_3_ antagonist, CAY10444 has only been shown to be selective in vivo at very low dosages which may not be enough to sufficiently block due to low affinity of the compound for the receptor [62]. Also, CAY10444 has been shown to inhibit [Ca^2+^]_i_ increases via purinergic P_2_ receptor or α_1A_-adrenoceptor stimulation and α_1A_-adrenoceptor-mediated contraction, while not affecting the S1P_3_-mediated decrease of forskolin-induced cAMP accumulation [63]. Inhibitors are not presently available for S1PR_4_ and S1PR_5_.

In the present study, FTY720 serves to demonstrate the potential clinical significance of targeting S1PR_1_ receptor activation in the inflammatory pain setting. It has been reported that blockade of the S1P-to-S1PR_1_ signaling pathway accounts for the observed beneficial effect of FTY720 in MS [64]. In support of this, recently developed S1PR_1_ antagonists, such as NIBR-0213, have been shown to have comparable therapeutic efficacy to FTY720 in models of MS [65]. As we show in this study, FTY720, like the S1PR_1_ antagonist W146, blocked carrageenan-induced thermal hyperalgesia and neutrophilic recruitment. In addition, FTY720 has been shown to be efficacious in the treatment of rheumatoid arthritis [66] and similar effects are observed with the S1PR_1_ antagonist, TASP0277308 [67]. Our work suggests that FTY720's clinical efficacy may extend into the chronic inflammatory pain setting, as in for the treatment of arthritis-induced pain.

While current and emerging therapeutics like NSAIDS and TRPV1 antagonists have been shown to have potent antinociceptive actions in the inflammatory pain setting, in part through their ability to block neutrophilic recruitment, adverse side effect profiles limit their viability as a long-term solution to chronic pain. Novel classes of drugs, such as those targeting S1P, whether used in combination with current analgesics or as a stand-alone treatment, may represent a novel approach in effectively treating chronic pain while avoiding unattractive side effects.

**Figure 7:**
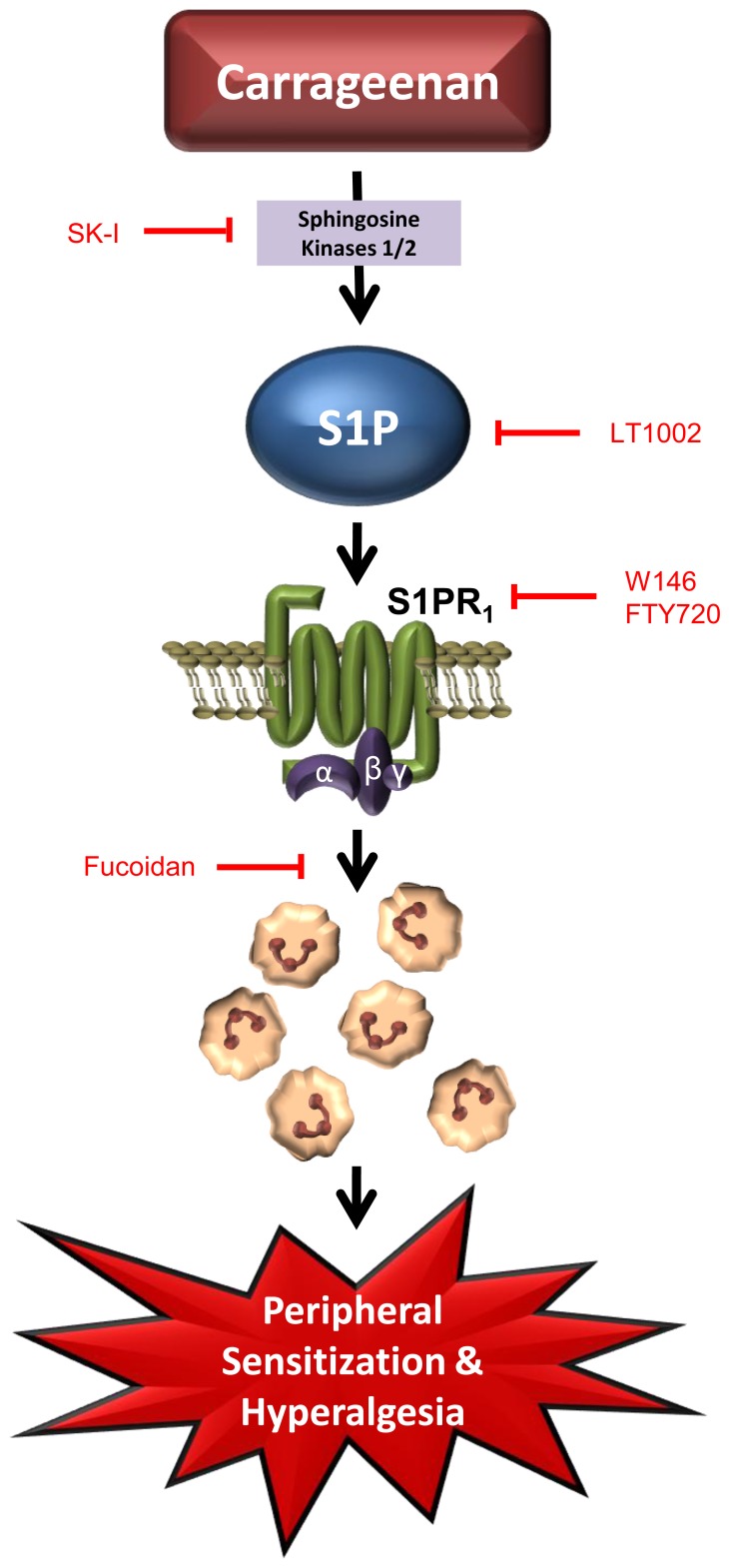
Schematic of proposed mechanisms behind S1P-mediated hyperalgesia. Carrageenan injection leads to the activation of sphingosine kinase enzymes favoring the conversion of sphingosine to bioactive S1P. S1P then goes on to activate S1PR_1_, initiating neutrophilic recruitment to the site of injury. Once there, neutrophils release several mediators known to sensitize nociceptors which induce peripheral sensitization and hyperalgesia.

In summary our findings show that S1P, through the activation of S1PR_1_, and the subsequent recruitment of neutrophils, plays a key role in inflammatory pain (summarized in Figure 7). Elucidating the mechanisms behind S1P's involvement in inflammatory pain can serve to identify targets for new therapeutic agents that may fill the void between NSAIDs and narcotics in the management of pain.

## References

[1] National CfHS (2006) Health, United States, 2006 Chartbook on Trends in the Health of Americans. Hyattsville, MD.

[2] Warner TD, Mitchell JA (2004) Cyclooxygenases: new forms, new inhibitors, and lessons from the clinic. FASEB J 18: 790–804. 15117884 10.1096/fj.03-0645rev

[3] Nixon GF (2009) Sphingolipids in inflammation: pathological implications and potential therapeutic targets. Br J Pharmacol 158: 982–993. 19563535 10.1111/j.1476-5381.2009.00281.x PMC2785521

[4] Ndengele MM, Cuzzocrea S, Masini E, Vinci MC, Esposito E, et al (2009) Spinal ceramide modulates the development of morphine antinociceptive tolerance via peroxynitrite-mediated nitroxidative stress and neuroimmune activation. J Pharmacol Exp Ther 329: 64–75. 19033555 10.1124/jpet.108.146290 PMC2670603

[5] Bryant L, Doyle T, Chen Z, Cuzzocrea S, Masini E, et al (2009) Spinal ceramide and neuronal apoptosis in morphine antinociceptive tolerance. Neurosci Lett 463: 49–53. 19631718 10.1016/j.neulet.2009.07.051 PMC2754041

[6] Muscoli C, Doyle T, Dagostino C, Bryant L, Chen Z, et al (2010) Counter-regulation of opioid analgesia by glial-derived bioactive sphingolipids. J Neurosci 30: 15400–15408. 21084596 10.1523/JNEUROSCI.2391-10.2010 PMC3000610

[7] Doyle T, Chen Z, Obeid LM, Salvemini D (2011) Sphingosine-1-phosphate acting via the S1P receptor is a downstream signaling pathway in ceramide-induced hyperalgesia. Neurosci Lett 499: 4–8. 21605625 10.1016/j.neulet.2011.05.018 PMC3119782

[8] Patti GJ, Yanes O, Shriver LP, Courade JP, Tautenhahn R, et al (2012) Metabolomics implicates altered sphingolipids in chronic pain of neuropathic origin. Nat Chem Biol 8: 232–234. 22267119 10.1038/nchembio.767 PMC3567618

[9] Kobayashi Y, Kiguchi N, Maeda T, Ozaki M, Kishioka S (2012) The critical role of spinal ceramide in the development of partial sciatic nerve ligation-induced neuropathic pain in mice. Biochem Biophys Res Commun 421: 318–322. 22503971 10.1016/j.bbrc.2012.03.153

[10] Salvemini D, Doyle T., Kress M., Nicol G. (2012) Therapeutic targeting of the ceramide-to-sphingosine-1-phosphate pathway in pain. Trends Pharmacol Sci: In Press. 10.1016/j.tips.2012.12.001 23318139

[11] Zhang YH, Vasko MR, Nicol GD (2006) Intracellular sphingosine 1-phosphate mediates the increased excitability produced by nerve growth factor in rat sensory neurons. J Physiol 575: 101–113. 16740613 10.1113/jphysiol.2006.111575 PMC1819432

[12] Doyle T, Finley A, Chen Z, Salvemini D (2011) Role for peroxynitrite in sphingosine-1-phosphate-induced hyperalgesia in rats. Pain 152: 643–648. 21239112 10.1016/j.pain.2010.12.011 PMC3039096

[13] Mair N, Benetti C, Andratsch M, Leitner MG, Constantin CE, et al (2011) Genetic evidence for involvement of neuronally expressed S1P receptor in nociceptor sensitization and inflammatory pain. PLoS One 6: e17268. 21359147 10.1371/journal.pone.0017268 PMC3040773

[14] Zhang YH, Fehrenbacher JC, Vasko MR, Nicol GD (2006) Sphingosine-1-phosphate via activation of a G-protein-coupled receptor(s) enhances the excitability of rat sensory neurons. J Neurophysiol 96: 1042–1052. 16723416 10.1152/jn.00120.2006

[15] Welch SP, Sim-Selley LJ, Selley DE (2012) Sphingosine-1-phosphate receptors as emerging targets for treatment of pain. Biochem Pharmacol 84: 1551–1562. 22971335 10.1016/j.bcp.2012.08.010

[16] Graler MH (2010) Targeting sphingosine 1-phosphate (S1P) levels and S1P receptor functions for therapeutic immune interventions. Cell Physiol Biochem 26: 79–86. 20502007 10.1159/000315108

[17] Hannun YA, Obeid LM (2008) Principles of bioactive lipid signalling: lessons from sphingolipids. Nat Rev Mol Cell Biol 9: 139–150. 18216770 10.1038/nrm2329

[18] Chi XX, Nicol GD (2010) The sphingosine 1-phosphate receptor, S1PR1, plays a prominent but not exclusive role in enhancing the excitability of sensory neurons. J Neurophysiol. 10.1152/jn.00709.2010 PMC2997035 20844107

[19] Xie W, Strong JA, Kays J, Nicol GD, Zhang JM (2012) Knockdown of the sphingosine-1-phosphate receptor S1PR1reduces pain behaviors induced by local inflammation of the rat sensory ganglion. Neurosci Lett 515: 61–65. 22445889 10.1016/j.neulet.2012.03.019 PMC3322267

[20] Snider AJ, Orr Gandy KA, Obeid LM (2010) Sphingosine kinase: Role in regulation of bioactive sphingolipid mediators in inflammation. Biochimie 92: 707–715. 20156522 10.1016/j.biochi.2010.02.008 PMC2878898

[21] Lee H, Lin CI, Liao JJ, Lee YW, Yang HY, et al (2004) Lysophospholipids increase ICAM-1 expression in HUVEC through a Gi- and NF-kappaB-dependent mechanism. Am J Physiol Cell Physiol 287: C1657–1666. 15294853 10.1152/ajpcell.00172.2004

[22] Lin CI, Chen CN, Lin PW, Lee H (2007) Sphingosine 1-phosphate regulates inflammation-related genes in human endothelial cells through S1P1 and S1P3. Biochem Biophys Res Commun 355: 895–901. 17331465 10.1016/j.bbrc.2007.02.043

[23] Xia P, Gamble JR, Rye KA, Wang L, Hii CS, et al (1998) Tumor necrosis factor-alpha induces adhesion molecule expression through the sphingosine kinase pathway. Proc Natl Acad Sci U S A 95: 14196–14201. 9826677 10.1073/pnas.95.24.14196 PMC24350

[24] Shimamura K, Takashiro Y, Akiyama N, Hirabayashi T, Murayama T (2004) Expression of adhesion molecules by sphingosine 1-phosphate and histamine in endothelial cells. Eur J Pharmacol 486: 141–150. 14975703 10.1016/j.ejphar.2003.12.022

[25] Tonnesen MG (1989) Neutrophil-endothelial cell interactions: mechanisms of neutrophil adherence to vascular endothelium. J Invest Dermatol 93: 53S–58S. 2666523 10.1111/1523-1747.ep12581069

[26] Bevilacqua MP, Stengelin S, Gimbrone MA Jr, Seed B (1989) Endothelial leukocyte adhesion molecule 1: an inducible receptor for neutrophils related to complement regulatory proteins and lectins. Science 243: 1160–1165. 2466335 10.1126/science.2466335

[27] Bennett G, al-Rashed S, Hoult JR, Brain SD (1998) Nerve growth factor induced hyperalgesia in the rat hind paw is dependent on circulating neutrophils. Pain 77: 315–322. 9808357 10.1016/S0304-3959(98)00114-6

[28] Cunha TM, Verri WA Jr, Schivo IR, Napimoga MH, Parada CA, et al (2008) Crucial role of neutrophils in the development of mechanical inflammatory hypernociception. J Leukoc Biol 83: 824–832. 18203872 10.1189/jlb.0907654

[29] Levine JD, Lau W, Kwiat G, Goetzl EJ (1984) Leukotriene B4 produces hyperalgesia that is dependent on polymorphonuclear leukocytes. Science 225: 743–745. 6087456 10.1126/science.6087456

[30] Levine JD, Gooding J, Donatoni P, Borden L, Goetzl EJ (1985) The role of the polymorphonuclear leukocyte in hyperalgesia. J Neurosci 5: 3025–3029. 2997412 10.1523/JNEUROSCI.05-11-03025.1985 PMC6565163

[31] O'Brien N, Jones ST, Williams DG, Cunningham HB, Moreno K, et al (2009) Production and characterization of monoclonal anti-sphingosine-1-phosphate antibodies. J Lipid Res 50: 2245–2257. 19509417 10.1194/jlr.M900048-JLR200 PMC2759830

[32] Hargreaves K, Dubner R, Brown F, Flores C, Joris J (1988) A new and sensitive method for measuring thermal nociception in cutaneous hyperalgesia. Pain 32: 77–88. 3340425 10.1016/0304-3959(88)90026-7

[33] Salvemini D, Wang ZQ, Wyatt PS, Bourdon DM, Marino MH, et al (1996) Nitric oxide: a key mediator in the early and late phase of carrageenan-induced rat paw inflammation. Br J Pharmacol 118: 829–838. 8799551 10.1111/j.1476-5381.1996.tb15475.x PMC1909531

[34] Bradley PP, Priebat DA, Christensen RD, Rothstein G (1982) Measurement of cutaneous inflammation: estimation of neutrophil content with an enzyme marker. J Invest Dermatol 78: 206–209. 6276474 10.1111/1523-1747.ep12506462

[35] Mullane KM, Kraemer R, Smith B (1985) Myeloperoxidase activity as a quantitative assessment of neutrophil infiltration into ischemic myocardium. J Pharmacol Methods 14: 157–167. 2997548 10.1016/0160-5402(85)90029-4

[36] Ley K, Linnemann G, Meinen M, Stoolman LM, Gaehtgens P (1993) Fucoidin, but not yeast polyphosphomannan PPME, inhibits leukocyte rolling in venules of the rat mesentery. Blood 81: 177–185. 7678063

[37] Semenov AV, Mazurov AV, Preobrazhenskaia ME, Ushakova NA, Mikhailov VI, et al (1998) Sulfated polysaccharides as inhibitors of receptor activity of P-selectin and P-selectin-dependent inflammation. Vopr Med Khim 44: 135–144. 9634715

[38] French KJ, Schrecengost RS, Lee BD, Zhuang Y, Smith SN, et al (2003) Discovery and evaluation of inhibitors of human sphingosine kinase. Cancer Res 63: 5962–5969. 14522923

[39] Delgado A, Casas J, Llebaria A, Abad JL, Fabrias G (2006) Inhibitors of sphingolipid metabolism enzymes. Biochim Biophys Acta 1758: 1957–1977. 17049336 10.1016/j.bbamem.2006.08.017

[40] Lee C, Xu DZ, Feketeova E, Kannan KB, Yun JK, et al (2004) Attenuation of shock-induced acute lung injury by sphingosine kinase inhibition. J Trauma 57: 955–960. 15580017 10.1097/01.ta.0000149495.44582.76

[41] Trifilieff A, Baur F, Fozard JR (2009) Role of sphingosine-1-phosphate (S1P) and the S1P(2) receptor in allergen-induced, mast cell-dependent contraction of rat lung parenchymal strips. Naunyn Schmiedebergs Arch Pharmacol 380: 303–309. 19636535 10.1007/s00210-009-0438-4

[42] Sanna MG, Wang SK, Gonzalez-Cabrera PJ, Don A, Marsolais D, et al (2006) Enhancement of capillary leakage and restoration of lymphocyte egress by a chiral S1P1 antagonist in vivo. Nat Chem Biol 2: 434–441. 16829954 10.1038/nchembio804

[43] Rosen H, Gonzalez-Cabrera P, Marsolais D, Cahalan S, Don AS, et al (2008) Modulating tone: the overture of S1P receptor immunotherapeutics. Immunol Rev 223: 221–235. 18613839 10.1111/j.1600-065X.2008.00645.x

[44] Rosen H, Gonzalez-Cabrera PJ, Sanna MG, Brown S (2009) Sphingosine 1-phosphate receptor signaling. Annu Rev Biochem 78: 743–768. 19231986 10.1146/annurev.biochem.78.072407.103733

[45] Russell FA, Fernandes ES, Courade JP, Keeble JE, Brain SD (2009) Tumour necrosis factor alpha mediates transient receptor potential vanilloid 1-dependent bilateral thermal hyperalgesia with distinct peripheral roles of interleukin-1beta, protein kinase C and cyclooxygenase-2 signalling. Pain 142: 264–274. 19231080 10.1016/j.pain.2009.01.021

[46] Brinkmann V, Davis MD, Heise CE, Albert R, Cottens S, et al (2002) The immune modulator FTY720 targets sphingosine 1-phosphate receptors. J Biol Chem 277: 21453–21457. 11967257 10.1074/jbc.C200176200

[47] Graler MH, Goetzl EJ (2004) The immunosuppressant FTY720 down-regulates sphingosine 1-phosphate G-protein-coupled receptors. FASEB J 18: 551–553. 14715694 10.1096/fj.03-0910fje

[48] Krump-Konvalinkova V, Yasuda S, Rubic T, Makarova N, Mages J, et al (2005) Stable knock-down of the sphingosine 1-phosphate receptor S1P1 influences multiple functions of human endothelial cells. Arterioscler Thromb Vasc Biol 25: 546–552. 15618544 10.1161/01.ATV.0000154360.36106.d9

[49] Lee OH, Kim YM, Lee YM, Moon EJ, Lee DJ, et al (1999) Sphingosine 1-phosphate induces angiogenesis: its angiogenic action and signaling mechanism in human umbilical vein endothelial cells. Biochem Biophys Res Commun 264: 743–750. 10544002 10.1006/bbrc.1999.1586

[50] Jia YT, Wei W, Ma B, Xu Y, Liu WJ, et al (2007) Activation of p38 MAPK by reactive oxygen species is essential in a rat model of stress-induced gastric mucosal injury. J Immunol 179: 7808–7819. 18025227 10.4049/jimmunol.179.11.7808

[51] Kulms D, Schwarz T (2006) NF-kappaB and cytokines. Vitam Horm 74: 283–300. 17027519 10.1016/S0083-6729(06)74011-0

[52] Vanden Berghe W, Vermeulen L, De Wilde G, De Bosscher K, Boone E, et al (2000) Signal transduction by tumor necrosis factor and gene regulation of the inflammatory cytokine interleukin-6. Biochem Pharmacol 60: 1185–1195. 11007957 10.1016/s0006-2952(00)00412-3

[53] Collins T, Read MA, Neish AS, Whitley MZ, Thanos D, et al (1995) Transcriptional regulation of endothelial cell adhesion molecules: NF-kappa B and cytokine-inducible enhancers. FASEB J 9: 899–909. 7542214

[54] Graves DT, Jiang Y (1995) Chemokines, a family of chemotactic cytokines. Crit Rev Oral Biol Med 6: 109–118. 7548618 10.1177/10454411950060020101

[55] Szabo C, Ischiropoulos H, Radi R (2007) Peroxynitrite: biochemistry, pathophysiology and development of therapeutics. Nat Rev Drug Discov 6: 662–680. 17667957 10.1038/nrd2222

[56] Salvemini D, Little JW, Doyle T, Neumann WL (2011) Roles of reactive oxygen and nitrogen species in pain. Free Radic Biol Med 51: 951–966. 21277369 10.1016/j.freeradbiomed.2011.01.026 PMC3134634

[57] Salvemini D, Jensen MP, Riley DP, Misko TP (1998) Therapeutic manipulations of peroxynitrite. Drug News Perspect 11: 204–214. 15616662

[58] Batinic-Haberle I, Cuzzocrea S, Reboucas JS, Ferrer-Sueta G, Mazzon E, et al (2009) Pure MnTBAP selectively scavenges peroxynitrite over superoxide: comparison of pure and commercial MnTBAP samples to MnTE-2-PyP in two models of oxidative stress injury, an SOD-specific Escherichia coli model and carrageenan-induced pleurisy. Free Radic Biol Med 46: 192–201. 19007878 10.1016/j.freeradbiomed.2008.09.042 PMC2742324

[59] Cuzzocrea S, Misko TP, Costantino G, Mazzon E, Micali A, et al (2000) Beneficial effects of peroxynitrite decomposition catalyst in a rat model of splanchnic artery occlusion and reperfusion. FASEB J 14: 1061–1072. 10834927 10.1096/fasebj.14.9.1061

[60] Salvemini D, Mazzon E, Dugo L, Riley DP, Serraino I, et al (2001) Pharmacological manipulation of the inflammatory cascade by the superoxide dismutase mimetic, M40403. Br J Pharmacol 132: 815–827. 11181422 10.1038/sj.bjp.0703841 PMC1572614

[61] Li C, Xie W-R, Strong J, Zhang JM, Nicol GD. JTE-013, a putative selective antagonist of sphingosine 1-phosphate receptor 2, increases the excitability of rat sensory neurons independently of the receptor 2011 2011; Washington, D.C. 10.1152/jn.00825.2011 PMC3544961 22673325

[62] Koide Y, Hasegawa T, Takahashi A, Endo A, Mochizuki N, et al (2002) Development of novel EDG3 antagonists using a 3D database search and their structure-activity relationships. J Med Chem 45: 4629–4638. 12361389 10.1021/jm020080c

[63] Jongsma M, Hendriks-Balk MC, Michel MC, Peters SL, Alewijnse AE (2006) BML-241 fails to display selective antagonism at the sphingosine-1-phosphate receptor, S1P(3). Br J Pharmacol 149: 277–282. 16940990 10.1038/sj.bjp.0706872 PMC2014271

[64] Chun J, Hartung HP (2010) Mechanism of action of oral fingolimod (FTY720) in multiple sclerosis. Clin Neuropharmacol 33: 91–101. 20061941 10.1097/WNF.0b013e3181cbf825 PMC2859693

[65] Quancard J, Bollbuck B, Janser P, Angst D, Berst F, et al (2012) A potent and selective S1P(1) antagonist with efficacy in experimental autoimmune encephalomyelitis. Chem Biol 19: 1142–1151. 22999882 10.1016/j.chembiol.2012.07.016

[66] Matsuura M, Imayoshi T, Okumoto T (2000) Effect of FTY720, a novel immunosuppressant, on adjuvant- and collagen-induced arthritis in rats. Int J Immunopharmacol 22: 323–331. 10689105 10.1016/s0192-0561(99)00088-0

[67] Fujii Y, Hirayama T, Ohtake H, Ono N, Inoue T, et al (2012) Amelioration of Collagen-Induced Arthritis by a Novel S1P1 Antagonist with Immunomodulatory Activities. J Immunol 188: 206–215. 22131329 10.4049/jimmunol.1101537

